# Spatial crime distribution and prediction for sporting events using social media

**DOI:** 10.1080/13658816.2020.1719495

**Published:** 2020-02-06

**Authors:** Alina Ristea, Mohammad Al Boni, Bernd Resch, Matthew S. Gerber, Michael Leitner

**Affiliations:** a Department of Geoinformatics, Doctoral College GIScience, University of Salzburg, Salzburg, Austria; b Boston Area Research Initiative, School of Public Policy and Urban Affairs, Northeastern University, Boston, MA, USA; c Product and Analytics, CyberCube, San Francisco, CA, USA; d Department of Geoinformatics, University of Salzburg, Salzburg, Austria; e Center for Geographic Analysis, Harvard University, Cambridge, MA, USA; f Department of Systems and Information Engineering, University of Virginia, Charlottesville, VA, USA; g Department of Geography and Anthropology, Louisiana State University, Baton Rouge, LA, USA

**Keywords:** Crime prediction, local kernel density estimation, violent tweets

## Abstract

Sporting events attract high volumes of people, which in turn leads to increased use of social media. In addition, research shows that sporting events may trigger violent behavior that can lead to crime. This study analyses the spatial relationships between crime occurrences, demographic, socio-economic and environmental variables, together with geo-located Twitter messages and their ‘violent’ subsets. The analysis compares basketball and hockey game days and non-game days. Moreover, this research aims to analyze crime prediction models using historical crime data as a basis and then introducing tweets and additional variables in their role as covariates of crime. First, this study investigates the spatial distribution of and correlation between crime and tweets during the same temporal periods. Feature selection models are applied in order to identify the best explanatory variables. Then, we apply localized kernel density estimation model for crime prediction during basketball and hockey games, and on non-game days. Findings from this study show that Twitter data, and a subset of violent tweets, are useful in building prediction models for the seven investigated crime types for home and away sporting events, and non-game days, with different levels of improvement.

## 1. Introduction

With massive social data available, research directions are spreading and changing views of politics, health, education, social and behavioral sciences. The popularity of social media and its contextual complexity facilitate the observation, analysis, and, occasionally, the prediction of human behavior based on the routine activities of the participants. The contextual complexity of social media data raises many obstacles when it comes to understanding human behavior patterns (e.g. people can talk about different ideas). The questions raised include how can we focus on the causality or explanation of the patterns in order to achieve a more error resistant prediction. While dealing with social media for crime analysis, the questions tackle what sort of social behavior can suggest the development of criminal behavior.

Crime occurrences are highly dependent on many factors, such as population distribution, socio-economic status, environmental components, weather, and citizen behavior during routine days and organized events. For example, during crowded event days, people’s routine activities vary slightly, which can lead to a short-term crime displacement (Marie 2016). Research shows that sporting events attract a high volume of people in specific activity nodes, such as sporting arenas or in pubs or bars to watch the games. These activity nodes can be criminogenic places, defined as crime attractors or generators (Brantingham and Brantingham 1993, 1995). In addition, a higher number of people use transportation routes that separate them from their normal routine trajectories. All these changes also have an influence on specific crime types because of fan behavior (Montolio and Planells 2016) or hooliganism (Caruso and Di Domizio 2013).

While considering Cohen and Felson’s theory of Routine Activities, which states that the coexistence of a motivated offender and a suitable target, and the absence of a possible guardian, increases the crime probability (Cohen and Felson 1979), we propose an event-routine activity, suggesting that spatial crime patterns are similar on event days and more dissimilar on non-event days. We follow a home-away game day versus non-game day (‘control day’) approach (Kurland 2014, Marie 2016, Montolio and Planells 2018). The spatial distribution of crime and associated tweets for home games is expected to show important changes in the spatial distribution, considering the attendance at the venue and the gathering of small crowds across the city or city establishments to watch the game. Away games may increase criminal activities throughout the city, considering that people gather in social areas (pubs, bars, restaurants) to watch the game together, while home games may increase crime occurrences around the arena as well. In addition, we argue for the usage of dynamic features in short-term spatiotemporal crime prediction models, such as the integration of location and semantic information from Twitter data.

An important research focus today is crime analysis and prediction using social media activity (Wang *et al*. 2012, Bogomolov *et al*. 2014). While the crime prediction models outlined in the literature include historical crime data, demographics, socio-economic, and built environment data as explanatory variables, this study proposes the integration of geo-located Twitter data and a subset of violent tweets as dynamic data for higher predictive accuracy. Although Twitter data was discussed in previous literature, based on geolocation or topic extraction (Gerber 2014, Al Boni and Gerber 2016), the evaluation of a violent subset in crime prediction models is novel. Our study confirms the importance of extracting valuable information from a high volume of data instead of using all the available data without understanding its complexity. Moreover, another important outcome considers analyzing the citywide patterns of crime occurrences and crime prediction models during two sporting events, basketball and hockey, in an enclosed venue using social media data.

The following two main hypotheses are tested in this research:

1. Spatial crime patterns have a different distribution when a sporting event occurs at a venue compared to control days, i.e. when there are no games at the stadium.
2. Geo-located Twitter messages and a subset of violent tweets improve crime prediction models for different crime types and enrich the information from historical crime data and additional explanatory variables.

## 2. Related work

### 2.1. Crime, social media and sporting events

Researchers have pursued spatial crime analysis for sporting events such as football in Europe (Caruso and Di Domizio 2013, Kurland 2014, Struse and Montolio 2014, Montolio and Planells 2016, 2018, Marie 2016, Kurland *et al*. 2017) and basketball (Yu *et al*. 2016), and for catastrophic events such as hurricanes (Curtis *et al*. 2006, Leitner and Helbich 2011, Leitner *et al*. 2011). In addition, similar types of events are strongly emphasized in social media text mining (Popescu and Pennacchiotti 2010, Fraustino *et al*. 2012, Corney *et al*. 2014, Hu 2014, Alqhtani *et al*. 2015, Lin 2015, Zhao *et al*. 2015).

Using a constructive dataset to represent a population or people’s locations during a specific timeframe is of high importance for crowd-based events. Integrating social media data in spatial crime analysis is done by considering Twitter messages’ locations as a proxy for ambient population and using this in crime rate calculations, thus, showing crime hotspots (Malleson and Andresen 2015b, 2016). Spatial crime analysis has been improved by incorporating a population density variable that is calculated using geo-coded social media messages (Malleson and Andresen 2015b, 2016, Kounadi *et al*. 2018), aggregated mobile phone counts (Botta *et al*. 2015, Malleson and Andresen 2016), human mobility data (Kadar *et al*. 2017), and population data from modeling such as Landscan (Andresen 2011, Kurland 2014, Malleson and Andresen 2015a).

### 2.2. Crime prediction using dynamic features

Spatiotemporal crime forecasting tools have received much attention in recent years from academics, private companies, law enforcement and police departments (Perry 2013). Traditionally, for crime prediction, historical crime data is used alone or together with crime attractors and generators (which can be demographic, environmental, and so on) in diverse types of prediction models (Caplan *et al*. 2011). For example, researchers used past crime data to predict burglaries by running different classifiers such as support vector machines (SVM), neural networks and Naïve Bayes (Yu *et al*. 2011), or self-exciting point process (SEPP) (Mohler *et al*. 2012). Others introduced demographic information from census blocks together with spatial data while using a General Additive Model (GAM) (Wang and Brown 2011, Ohyama and Amemiya 2018). However, these additional variables are constant with low changes over time and do not account for the dynamic occurrences of crimes. Location-based services (LBS), including social media, have become widespread in recent years and they include spatial and temporal dynamic variability, which can be a valuable addition to traditional prediction models. The availability of ‘big data’ helps bridging the gap between low and high computational models for crime (Zhao and Tang 2018).

Crime prediction models in conjunction with social media data have been able to achieve a significantly better rate of success for certain crime types, compared to traditional crime prediction models (Gerber 2014, Al Boni and Gerber 2016). Machine learning techniques together with linear and logistic modeling (Wang *et al*. 2012, Alruily 2012, Wang and Gerber 2015, Burnap and Williams 2015), density based models (Featherstone 2013b, 2013a, Bendler *et al*. 2014a, Cheng and Smyth 2015, Al Boni and Gerber 2016b, Hu *et al*. 2018), risk terrain modeling (Perry 2013), and Geographically Weighted Regression (Bendler et al. 2014b, Ristea *et al*. 2018, Ohyama and Amemiya 2018) have been used to predict crime occurrences.

Many of the models are theory-driven, mostly from environmental criminology (Caplan *et al*. 2011). Most targeted prediction models use classification strategies (predicting crime (1) or no crime (0)), while fewer researchers are considering crime incident counts. For example, Vomfell and colleagues built a multi-model solution for predicting the number of crime incidents per census tracts by combining demographic, social media, and taxi flow data, showing that dynamic variables influence prediction of property crime more than of violent crime (Vomfell *et al*. 2018).

The aim of recent research has been short-term crime occurrence prediction using human behavior and mobility data from diverse mobile networks. Check-ins from Foursquare, local search and recommender mobile app are used to calculate visitor entropy, region popularity and other parameters to be later introduced in the prediction models (Kadar *et al*. 2016, Zhao and Tang 2017, Rumi *et al*. 2018). Moreover, besides check-in information, researchers have introduced data about pick-up and drop-off from taxi flows and regional Point of Interest (POI) data (Wang *et al*. 2016), as well as subway data and other static information (Kadar and Pletikosa 2018). Recently, Yang et al. released CrimeTelescope, the first online system for crime hotspots prediction, which fuses static urban information (demographics) with POI information from Foursquare and social media (i.e. Twitter) (Yang *et al*. 2018).

However, previous works has not studied the effect of sporting events and their impact on crime likelihood by adding dynamic features to prediction models. Dynamic information is accessible nowadays, and it is important to integrate it into crime prediction models together with information about changes in the city, such as public events occurring.

## 3. Data and methods

We present a spatial crime analysis in the city of Chicago, the home location of the Chicago Bulls of the National Basketball Association (NBA) and the Chicago Blackhawks of the National Hockey League (NHL). The analyzed period contains two seasons, 2012–2013 and 2013–2014. The United Center, where the home games of these two teams take place, is the largest such venue in the United States and hosts many types of events per year, including concerts, family events, television and political events (United Center 2018). On average, 21,776 and 22,623 fans attended Blackhawks games in the 2012–2013 and 2013–14 seasons, respectively (HockeyDB 2018), while 21,876 and 21,716 attended the average Bulls game in those years (ESPN 2018). Crime and geo-tagged Twitter data for all home and away game days for both teams, together with control days, were collected. Tweets are semantically analyzed, and a subset of violent tweets is extracted for the same categories mentioned above. The analysis is based on a 200m x 200m cell size grid applied to the City of Chicago. We define five temporal subsets, called ‘bins’ in the rest of the text, according to the two sports teams playing at the United Center venue: home games for Bulls; away games for Bulls; home games for Blackhawks; away games for Blackhawks; and control days. Seven crime types are analyzed, three violent crime types (robbery, assault and battery) and four property crime types (criminal damage, motor vehicle theft, other offense (such as telephone threat and harassment, other vehicle offense, violate order of protection), and theft). Spatial prediction models from this study include demographic, socio-economic and environmental variables. In order to define the main features contributing to higher crime prediction, we use a random forest classifier. Although certain features appear to be good crime predictors, it does not imply a causal effect. Such findings are important, though, and provide useful insights for crime analysts.

### 3.1. Data

In summary, this study uses crime occurrences, Twitter data, and demographic, socio-economic and environmental data about the city of Chicago. Each source is described in more detail in the following sections, together with the applied preprocessing steps.

The city of Chicago offers an open data portal, which includes a multitude of freely available information about the city regarding administration and finance, buildings, community, education, environment, events, public safety, sanitation and transportation (City of Chicago 2018). It is possible to download reported crime incidents as of 2001 and up until the most recent seven days using the portal. These incidents are extracted from the Chicago Police Department’s CLEAR (Citizen Law Enforcement Analysis and Reporting) database; the crime location data considers geo-privacy concerns (e.g. appropriate geographic masking is applied to preserve geospatial privacy). Also, it is worth mentioning that crime data have their own shortcomings (Quillian and Pager 2001) due to under-reporting to the police (e.g. due to a fear of reporting crimes, less serious crimes are less frequently reported) or other reasons.

In this study, we extract 111,936 incidents that occurred between 10/31/2012 and 04/14/2014 (only for specific dates as further discussed in Section 3.3). We extract the latitude-longitude locations, the timestamp, and the crime type of each incident. The aggregated crime data covers 30 crime types. Patterns for crime types are demonstrated to be different in time and space (Andresen and Linning 2012), so this study shows results for aggregated and disaggregated crime types. For the disaggregated crime types, we focus on seven types of crime, which together account for ~70% of all crimes. Table 1 depicts the per-type frequencies of these crimes. These types are selected because they are some of the most prevalent in Chicago (narcotics is another prevalent crime type that is not analyzed in this study).

**Table 1.** Reported crime records in Chicago, Illinois, US Chicago police department’s CLEAR (counts for the five bins used in this study) *relative total includes just the seven crime types considered in this study; the real value includes 30 crime types.

Crime Type	Frequency
Assault	6,446 (8.32%)
Battery	18,875 (24.35%)
Criminal damage	11,010 (14.20%)
Motor vehicle theft	4,837 (6.24%)
Other offense	6,978 (9.00%)
Robbery	4,046 (5.22%)
Theft	25,313 (32.66%)
Relative Total*	77,505 (100%)

Researchers show the importance of considering the built environment in spatial crime analysis (Kinney *et al*. 2008, Grubesic and Pridemore 2011, Groff and Lockwood 2014), so we extract spatial features from the same data portal to characterize the physical environment: hospitals, parks, bike racks, liquor stores, bars, restaurants, major streets, neighborhoods, rail roads, pedestrian streets, pedestrian ways, police stations, Chicago Transit Authority (CTA) stations, CTA bus stops, CTA rail lines, CTA routes, safe passages, and schools. The complexity of crime characteristics in space and time, and all the factors that may be crime attractors, generators, or other explanatory variables, influence the displacement and the dynamic behavior of crime occurrences (Braga 2005, Braga and Bond 2008, Braga *et al*. 2014).

We extract demographic and socio-economic data at the census tract spatial resolution (Manson *et al*. 2018, City of Chicago 2018). In order to disaggregate the information for the unit of analysis in this study, we use the function ‘summarize within’ in ArcGIS (ESRI 2018). Besides the housing information from the Census, we also add information about Airbnb locations and prices to complement the residential information (Center for Spatial Data Science 2018).

The most widely applied population statistic in crime analysis is the residential population. The population distribution from the census is collected at different spatial scales (e.g. neighborhoods, grid cells, or city level). However, the residential population is an inappropriate statistic for mobile crime types (e.g. street robbery), so there is a need for ambient dynamic population data. Researchers suggest different ways of calculating the ambient population for crime analysis (Zhang *et al*. 2012, Malleson and Andresen 2015b, 2016, Kounadi *et al*. 2018). The LandScan global population model, which represents the ambient population, is modeled by the Oak Ridge National Laboratory using spatial data, imagery analysis, and a multi-variable dasymetric modeling approach to disaggregate census counts within administrative boundaries (Bright *et al*. 2016). In this paper, we create population at crime risk (ambient) models (resolution 200m x 200m cell size) for each of the five bins by using LandScan data as the source zones and Twitter data as ancillary points, following the model and code proposed by (Kounadi *et al*. 2018). The population at crime risk or at risk of falling victim to a crime is an explanatory variable complementing the Census data.

Finally, tweets are extracted using the Twitter API (Twitter Inc 2018). Only geo-tagged tweets are considered since the purpose of this study requires geospatial, temporal, and semantic analysis. All tweets can serve as a proxy for general online *activity* in the city of Chicago. No text filter is applied when extracting the Twitter data. Several practical questions may arise because the geographically located tweets represent no more than 5% of all tweets posted online (Zhang *et al*. 2016). However, GPS has much better spatial and temporal quality compared to other localization approaches such as cell-towers. Therefore, tweets might be less representative but more accurate. We use a bounding box filter within the Twitter API, defining the upper-right corner as −87.52413, 42.02303 and the lower-left corner as −87.94011, 41.64454. We collect 9,436,276 GPS-tagged tweets authored by 644,514 different users within Chicago between 10/31/2012 and 04/14/2014. In addition to GPS coordinates, Twitter’s API provides each tweet with a timestamp and textual content.

Online databases (Gracenote 2018, Sportradar 2018) are used to extract home and away game days for the basketball and the hockey team. In this research, we select the entire day in which a game was played, without considering the start and the end times of games. This means we use 24 hours of aggregated crime and Twitter data and we are discussing daily patterns. Criminal behavior may change across day/night time periods for specific crime types; however, we do not focus on those differences in this paper.

### 3.2. Methods

The purpose of this study is to investigate how spatial crime distribution is influenced by sporting games at the United Center venue and the correlation between crime occurrences and the density of geo-located Twitter data. In addition, the study explores the impact of tweets as a possible crime predictor.

Because of the limited time between the hockey and basketball games, we have difficulties in finding a reasonable subset of data that was comparable. We do not include days in which one of the teams has a home game and the other an away game, or when both teams are playing away in this analysis. This is done to partially avoid misunderstandings in the patterns, such as changes regarding a lost away game for basketball while there is a home hockey game – it would be difficult to define whether (any) changes occur for a particular reason. These issues lead us to reduce the data to 30 days for each bin. We define the control days in relation to the home and away games. Namely, we select similar days of the week in the same month where no sporting event occurred at the stadium. Table 1 shows the number of crimes per crime type used in this study. The control period for game days is on the same day of the week and at the same time of the year (Brimicombe and Cafe 2012). If that is not possible, the control is a similar day of the week (Table 2). Week days are considered to be Monday to Thursday and weekend days Friday to Sunday – normally, Friday is not a weekend day, however, according to criminal patterns for some crime types it is more relevant to consider it as a weekend day (Brimicombe and Cafe 2012).

**Table 2.** Day selection for the five bins.

	Bulls		Blackhawks		
	home	away	home	away	Control days
Mon-Thu	20	19	17	20	19
Fri-Sun	10	11	13	10	11

Furthermore, we determine a method of extracting ‘violent messages’, tweets where users are using violent words, hate words or swear words. Firstly, we use a joined lexicon of hate-related terms from hatebase (Hatebase 2018) – a repository of crowdsourced speech terms (Gao *et al*. 2017, Davidson *et al*. 2017) and noswearing (No swearing 2018) – a crowdsourced database of swear words (Founta *et al*. 2018). We extract all the geo-located tweets that contain at least one word from these predefined dictionaries previously used in literature. Hate speech and sentiment analysis have a close relationship, with several approaches showing the importance of using sentiment analysis as auxiliary classification (Schmidt and Wiegand 2017). Thus, secondly, we apply sentiment analysis by using the NRC lexicon (Mohammad and Turney 2010, 2013), after which we extract only the negative messages. The resulting subset is used as ‘violent tweets’ – different subsets according to the bins. The messages can contain offensive language; include hate crime elements or other negative connotations. Thus, we assume that a daily ‘violent’ tweet hotspot is helpful to predict daily crime hotspots. In addition, we expect to have more ‘violent’ tweets during sporting events than during comparison days, and by their spatiotemporal relationship with crime occurrences, it will improve prediction.

**Figure 1.**
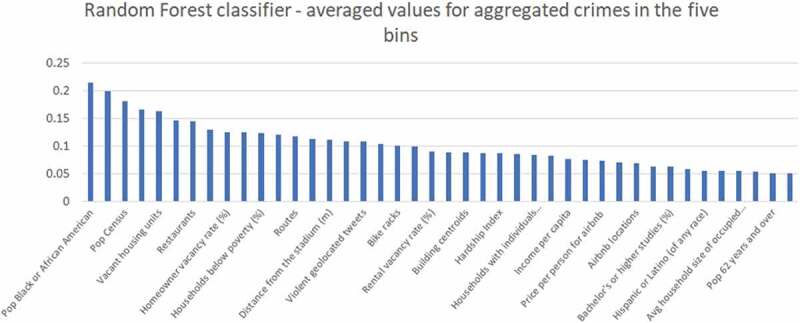
Feature selection using random forest.

As shown in previous literature, many types of data can potentially contribute to prediction accuracy in appropriate contributions. However, by having an increased number of features, we can encounter more noise in the data. Thus, we address the problem of feature selection in order to derive a significant size and improve prediction while avoiding noisy or irrelevant data. We use the library ‘party’ in R, which is an implementation of the random forest and bagging ensemble algorithms utilizing conditional inference trees as base learners. By applying the function ‘cforest’, we obtain the importance of each value in the dataset with crime type as the dependent variable. We eliminate the features with importance coefficient below 0.05. We did not find a clear statement about calculating a specific threshold, thus we selected the value from which the importance shows a growing path. Figure 1 shows the most important features that will be used in the analysis.

**Figure 2.**
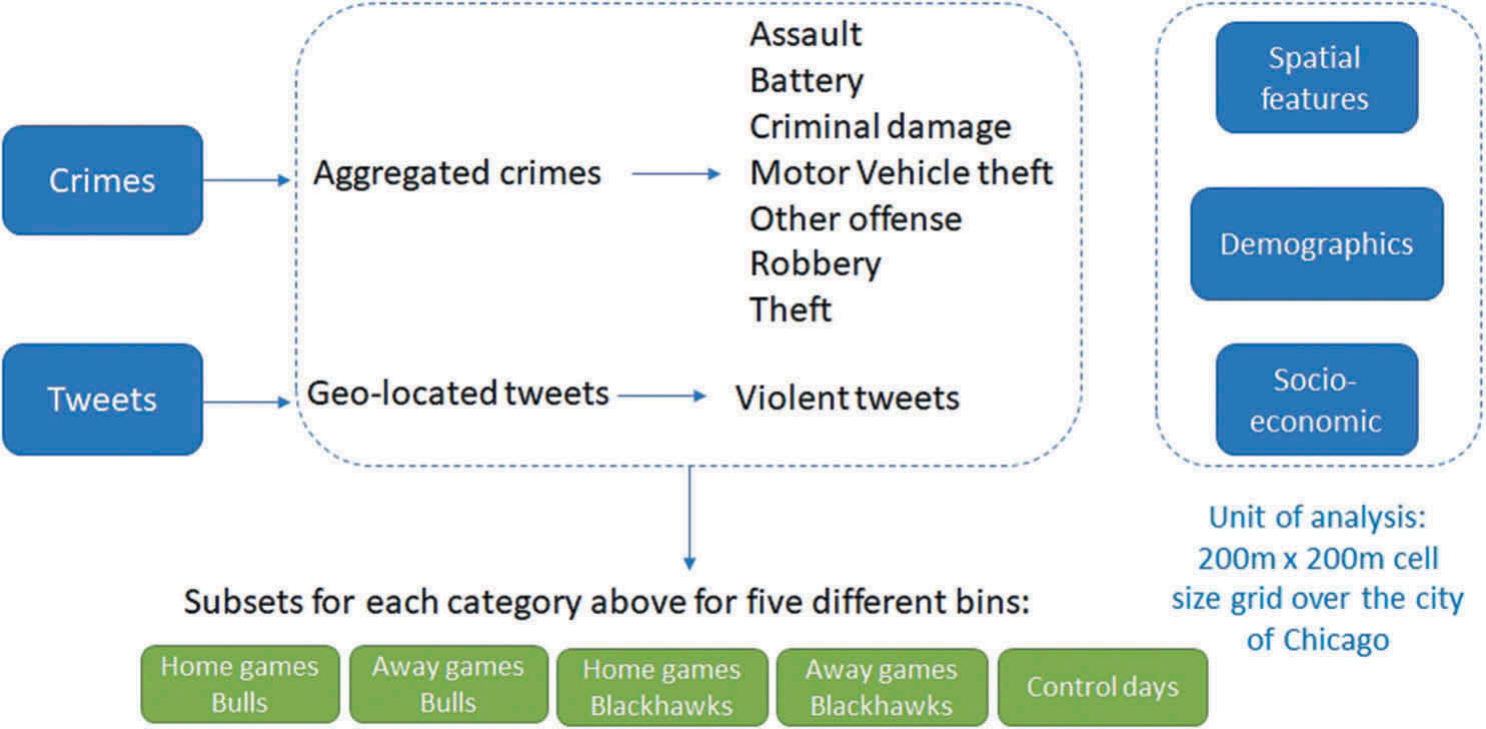
Data used in this case study.

Finally, all data subsets and additional features are clipped for the study area and aggregated to 200m x 200m cell size of a regular grid, which is superimposed over the city of Chicago. Grids with the same resolution are frequently used in criminological research (Gerber 2014, Hoeben *et al*. 2014, Al Boni and Gerber 2016b, Al Boni and Gerber 2016a, 2016c, Rummens *et al*. 2017). Figure 2 shows a schema of the data used in our analysis, while Table 3 shows the predictor variables used in this study.

**Table 3.** Summary of predictor variables used in this analysis.

Crime history variables:
Historical crime data before prediction day for the five bins
Demographic variables:
Population at crime risk (different for the five bins), residential population; population white, population black or African American, population Asian, population 62 years and over, foreign-born (%), 25 years and over high school or General Educational Development, total 25 years and over, 25 years and over less than high school, 25 years and over some college, foreign-born, household with individuals under 18 years, population 18 years and over total, households by type: non-family, households by type: husband-wife family, Bachelor’s or higher studies (%), 25 years and over bachelor’s degree or higher, Hispanic or Latino (of any race), average household size of occupied housing units by tenure: owner-occupied, average household size of occupied housing units by tenure: renter-occupied, median age by sex for both sexes
Socio-economic variables:
Vacant housing units, homeowner vacancy rate (%), unemployed, households below poverty (%), below the poverty level (%), rental vacancy rate (%), occupied housing units, hardship index, income per capita, the price per person for Airbnb, Airbnb locations
Environmental variables:
restaurants, bars, bus stops, buildings, bike racks, transportation routes: density
stadium: distance
Dynamic variables:
Geo-located Twitter data for the five bins: density and distance
Violent Tweets for the five bins: density and distance

#### 3.2.1. Spatial distribution, correlation, and regression analysis

To determine the spatial distribution of crime, tweets, and violent tweets, density maps are created with the 200m x 200m cell size as the base unit (a total of 15,574), and the days from each bin aggregated per grid cell – represented in map pie charts (Figures 5–7). Considering the methodological framework and the purpose of showing differences during game days and control days, we present density maps for a zoom-in analysis within a 1 km buffer around the stadium because this distance around the stadium suggests an elevated risk of offenses during game days (Kurland *et al*. 2014).

In order to study the spatial relationship between crime and tweets, we use the Local Indicators of Spatial Association (LISA) approach in two different ways. First, we test each of the crime and tweets datasets for spatial autocorrelation and calculate the global Moran’s I values (Formula 1). Second, we use the bivariate spatial correlation statistic and calculate bivariate Moran’s I values (Formula 2) for each of the bins as input data and each of the tweets datasets as lagged data (Anselin 1995, Anselin and Kelejian 1997, Anselin *et al*. 2006). We present spatial autocorrelation indexes for the city of Chicago and for a zoom-in analysis within a 1 km buffer around the stadium. A spatially lagged variable (a sum of spatial weights multiplied with values for observations at neighboring locations) is essential for spatial autocorrelation analysis. Thus, for the bivariate case in this study, the y-axis pertains to neighboring values for tweets or violent tweets, while the x-axis considers the locations of crimes.

$$\;\;\;\;\;\;\;\;\;\;\;\;\;\;\;\;\;\;\;\;\;\;\;\;\;\;\;\;\;\;\;\;\;\;\;\;I=\frac{\sum_{i=1}^{n}\sum_{j=1}^{n}w_{ij}z_{i}z_{j}/S_{0}\;}{\sum_{i}z_{i}^{2}/n}\;\;\;\;\;\;\;\;\;\;\left(Formula\;1\right)$$

where *n* is the number of observations, $S_{0}=\underset{i}{\sum }\underset{j}{\sum }w_{ij}$ as the sum of all the weights, and $w_{ij}$ as the elements of the spatial weights matrix.

$$\;\;\;\;\;\;\;\;\;\;\;\;\;\;\;\;\;\;\;\;\;\;\;\;\;\;\;\;\;\;\;\;I_{BV}=\frac{\sum_{i}\left(\sum_{j}w_{ij}*x_{i}\right)}{\sum_{i}x_{i}^{2}}\;\;\;\;\;\;\;\;\;\;\;\;\;\;\;\left(Formula\;2\right)$$

where values are similar to (Formula 1), with the exception of $x_{i}$ which is the lagged variable value at a particular location. It can also be considered the slope of a regression of $W_{y}$ on x, where x is the explanatory variable and $W_{y}$ is the spatial lag of the dependent variable (all variables are standardized, and the spatial weights are row standardized).

#### 3.2.2. Spatial prediction

We adopt our crime prediction models from Gerber (2014). This model treats crime prediction as a classification problem, where the units of classification are spatial points p and the response is binary for the first part of modeling (see chapter 4.2), indicating the odds of observing a crime at point p. In other words, the model estimates the relative risk of crime type T at point p using a set of predictor features. To build our crime prediction models, we first discretize the geospatial surface of an area of interest. We create a grid of points with a fixed cell size. Each of these points is labeled *NONE* (for the non-occurrence of crime). Then, we create points from the locations of all known crimes of type *T* and combine these points with the *NONE* points. In cases where a *NONE* and *T* point coincide, we remove the former. Next, we use all points (*NONE* and *T* points) to train a binary classifier with the following form (Formula 3):

$$\;\;\;\;\;\;\;\;\;\;\mathrm{Pr}\left(Label_{p}=T|f_{1}\left(\theta_{p}\right),\ldots ,f_{n}\left(\theta_{p}\right)\right)=\frac{1}{1+e^{-\left(\beta_{0}+\sum_{i=1}^{n}\beta_{i}*f_{i}\left(\theta_{p}\right)\right)}}\;\;\;\;\left(Formula\;3\right)$$

where $f_{1}\left(\theta_{p}\right),\ldots ,f_{n}\left(\theta_{p}\right)$ are features describing point p with parameters $\theta_{p}$. In other words, our model contains coefficients representing the relationship between (1) crime occurrence and non-occurrence at point p and (2) various features of p. These coefficients apply uniformly to the entire study region. This formulation allows for building a wide range of models by quantifying appropriate features. We use two types of spatial density features, including kernel density estimation (KDE), and localized kernel density estimation (LKDE). The KDE is formally defined in Formula 4:

$$\;\;\;\;\;\;\;\;\;\;\;\;\;\;f\left(\theta_{p}=\left\{p\right\}\right)=k\left(p,h\right)=\frac{1}{Ph}\overset{P}{\underset{j=1}{\sum }}K\left(\frac{||p-p_{j}||}{h}\right)\;\;\;\;\;\;\;\;\;\;\;\;\left(Formula\;4\right)$$

where P is the total number of spatial points (e.g. crime incidents, tweets, location of bus stops, etc.), h is a smoothing parameter (bandwidth), p is the point at which a density estimate is calculated, ||·|| is the L-2 norm, and K is an interpolation (kernel) function. The KDE method is frequently used for hotspot mapping. It is one of the most widely used methods in spatial crime analysis, along with other hot spot techniques such as Gi*, choropleth mapping. For KDE, three parameters need to be set: grid cell size, interpolation method (kernel function), and search radius (bandwidth). We used the kde function from the ks package in R, optimizing the bandwidth with the Hpi heuristic for the 200m x 200m cell size.

The localized version (i.e. LKDE algorithm; introduced in (Al Boni and Gerber 2016b, 2016c) uses data-driven localized estimators to produce non-smooth density estimates. LKDE parameters are automatically optimized using a genetic algorithm: In LKDE, the interpolation method can vary with respect to each cell depending on the kernel weight. The approach requires decisions about kernel size and convolution values. In addition, LKDE computational power is faster than KDE. In order to create the density estimate, the LKDE process involves building an overlay grid, counting the incident frequency per grid cell, fixing the center of a convolutional kernel in each cell and performing a convolutional operation. Therefore, we chose to use LKDEs to estimate historical crime density features and KDEs for the remaining spatial features. Such features (e.g. restaurants, bars) estimate the spatial density of entities at p, as measured by yet another KDE (e.g. the value at p of the KDE built from police station locations). We use LibLinear to estimate coefficients within the logistic regression model (Fan *et al*. 2008, Gerber 2014). This is an open source library for large-scale classifications, supporting logistic regression and linear support vector machines, from which we use L2-regularized logistic regression. Crime occurrences are concentrated in a few hot areas and sparse in others, thus making the training set very imbalanced. Thus, the classifier tends to over-predict the absence of crime. In order to cover class imbalance (more grid cells will be empty compared with the ones including crimes), we set LibLinear to negative/positive counts of points in the training set. The prediction results consist in probabilities, which are then used in the evaluation methods.

**Figure 3.**
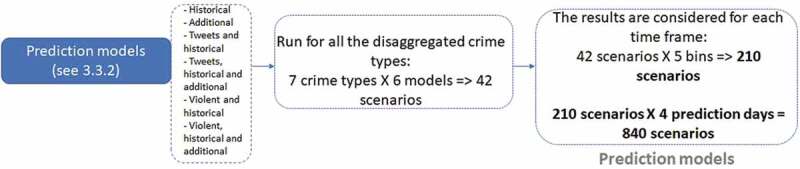
Prediction models design.

Next, we present six models including various subsets of features mentioned above. It is worth mentioning that for this study design we can use two different parameters for the predictors: density and/or distance. Distance features indicate the linear distance from an analysis point to the spatial entities, whereas density features quantify the spatial density of those entities at that point. We chose the density and distance-based measures for the tweets and violent tweets, instead of choosing one parameter. We are planning to discuss the difference between parameter choices in future work. Figure 3 shows these models for a fair understanding.

1. **Historical Crime Density Model (Historical)** is a model that includes only an LKDE feature of historical crime records.
2. **Historical Crime and Additional Data Model (Additional and historical)** is a model that adds to the Historical model the additional density features (see Section 2.2 Data for a detailed description of these features) that characterize the demographical, socio-economic, and physical environment in Chicago.
3. **Historical Crime and Tweets Model (Tweets and historical)** is a model that adds to the Historical model a KDE feature, estimated from all geo-tagged tweets from the same days as crimes.
4. **Historical Crime, Additional, and Tweets Model (Tweets, historical and additional)** is a model that adds the same additional features (see Section 3.2 Data for a detailed description of these features) as above to the previous model.
5. **Historical Crime and Violent Tweets Model (Violent tweets and historical)** is a model that adds to the Historical model a KDE feature estimated only from violent geo-tagged tweets from the same days as crimes.
6. **Historical Crime, Additional, and Violent Tweets Model (Violent tweets, historical and additional)** is a model that adds the same additional features (see Section 3.2 Data for a detailed description of these features) as above to the previous model.

#### 3.2.3. Evaluation methods

**Figure 4.**
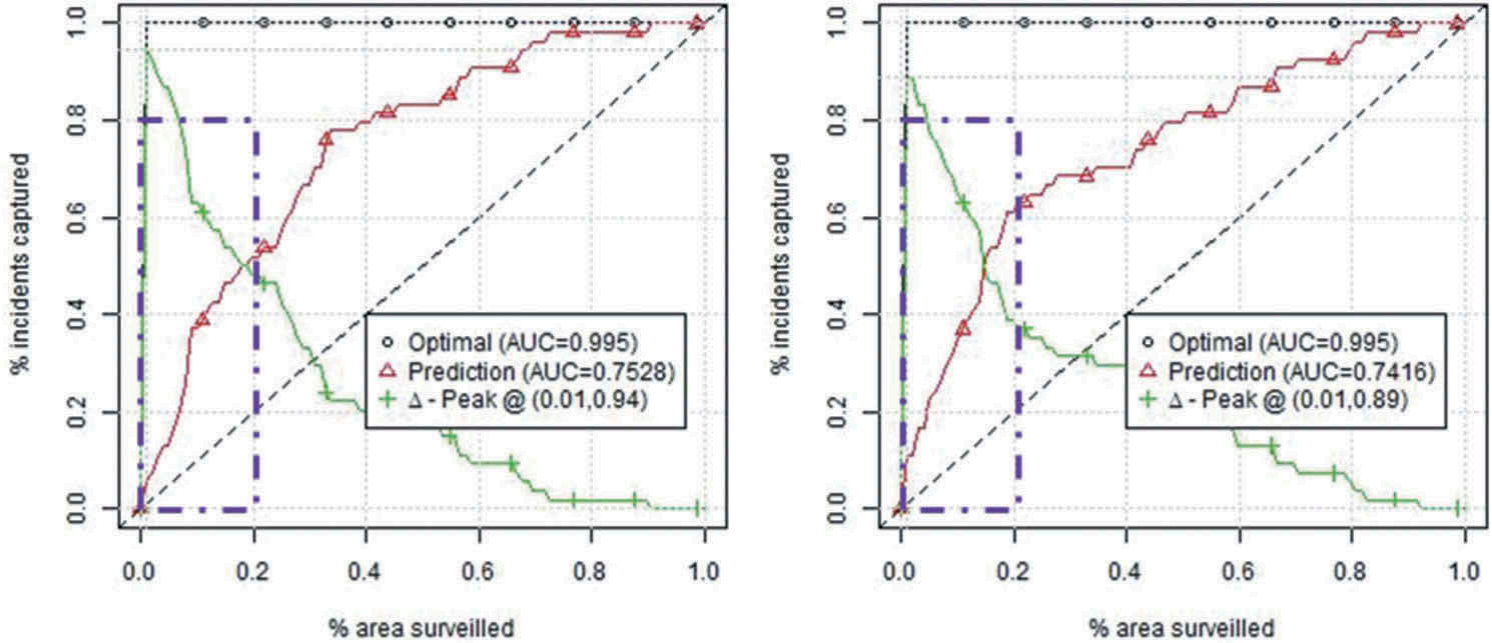
Example of surveillance plots for other offense for two different prediction days, home games Chicago Bulls.

We evaluate the performance of prediction models using the Area under the Curve (AUC) of surveillance plots (Gerber 2014). These plots show the proportion of true future crimes (y-axis or sensitivity) that occur in a percentage of the most threatened area predicted by the model (x-axis). AUC is calculated as a summary of the surveillance plot (Figure 4). Researchers have discussed a number of evaluation models for crime prediction (Chainey *et al*. 2008, Levine 2008, Gerber 2014), a well-known one being the Predictive Accuracy Index (PAI), which is calculated as the hit rate (ratio of incidents occurring in the hotspots divided by the total number of occurrences) divided by the ratio between the total area of predicted crime and total area of study (Chainey *et al*. 2008). Surveillance plots generalize the PAI to include prediction performance for the total area analyzed. The interest is twofold: first, to have curves that approach the upper left corner: the example below shows that in the top 20% of the most threatened area of the city of Chicago, 50% of the real incidents are captured; second, to have curves with higher AUC scores, which shows better performance for the entire city.

The AUC measures the accuracy of a quantitative test, and it has values between 0 and 1.0 (a good classifier should have a higher AUC than 0.5). In this study, we do not apply the area under the receiver operating characteristics (ROC) curve in its initial form (Fawcett 2006).

## 4. Results

### 4.1. Spatial crime and tweets distribution and correlation

**Figure 5.**
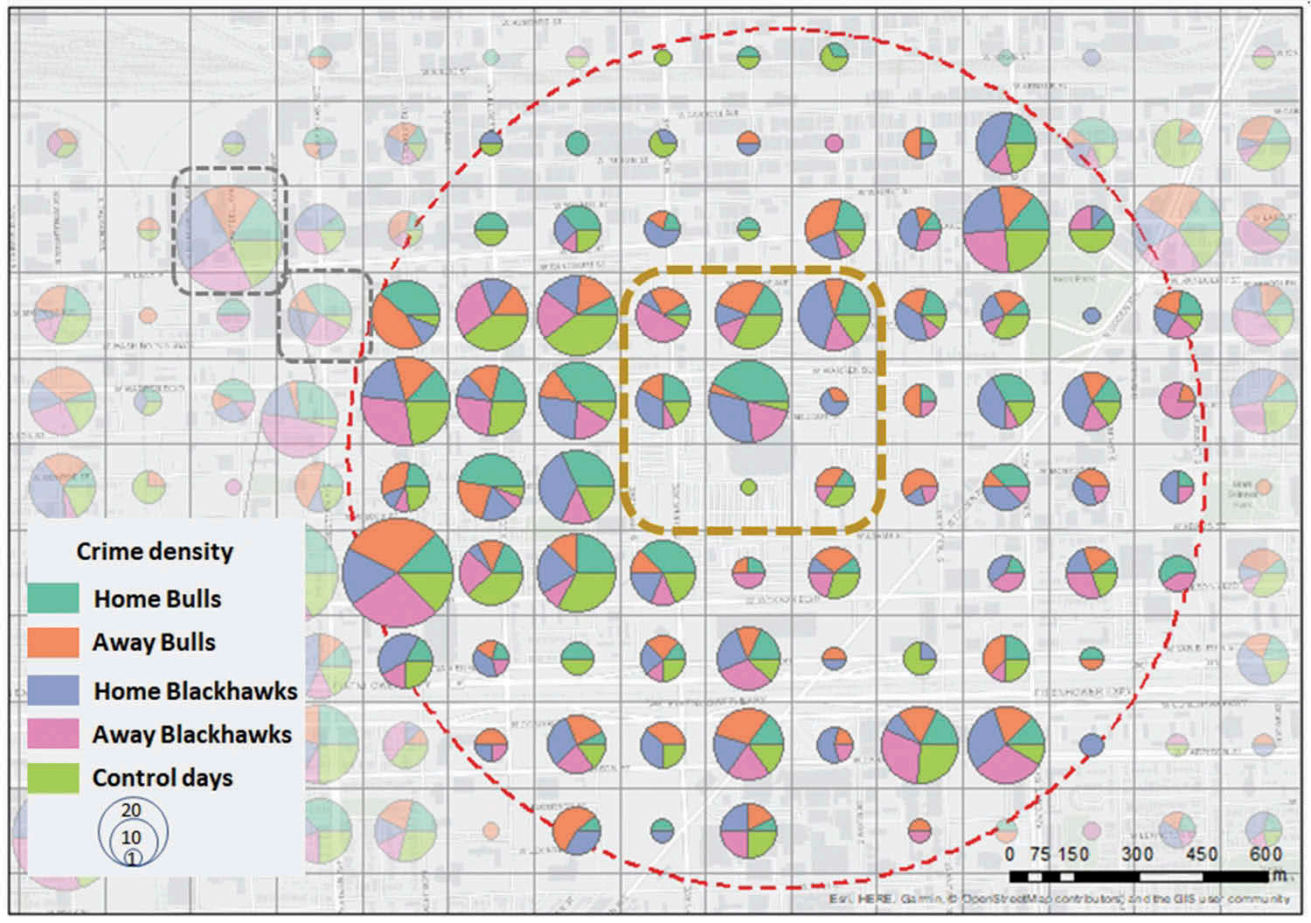
Density distribution of crimes around the venue, where gray squares represent areas with similar crime densities, and brown square with higher crime density during game days; red dots circle is the 1km buffer around the venue.

We analyzed crime and tweets density for a one-kilometer buffer around the United Center for the five bins. Crime densities showed mixed information for the area around the stadium (Figure 5). In one of the arena’s grid cells, most of the crimes occur during home game days, 11 of them for Bulls and 8 of them for Blackhawks. We noticed that near the southern part of the stadium, only one crime was reported during the home games for the Bulls. This might be related to police recordings: When a crime occurs at the stadium, it is recorded in a specific location that can take place at one of the stadium entrances. We noticed more crime occurrences in the grid cell above the southern part of the stadium. In many grid cells in the west and southwest parts of the stadium, the crime occurrences have high rates in all five bins – showing clear hotspots regardless of the events in close proximity. Yet, there is a different distribution of *specific* crime types. Criminal damage occurrences happen more during home games of the Bulls than during home games of the Blackhawks. Other offenses occur more frequently during home games of the Blackhawks. Motor vehicle theft shows similar occurrences for both Bulls and Blackhawks home games.

**Figure 6.**
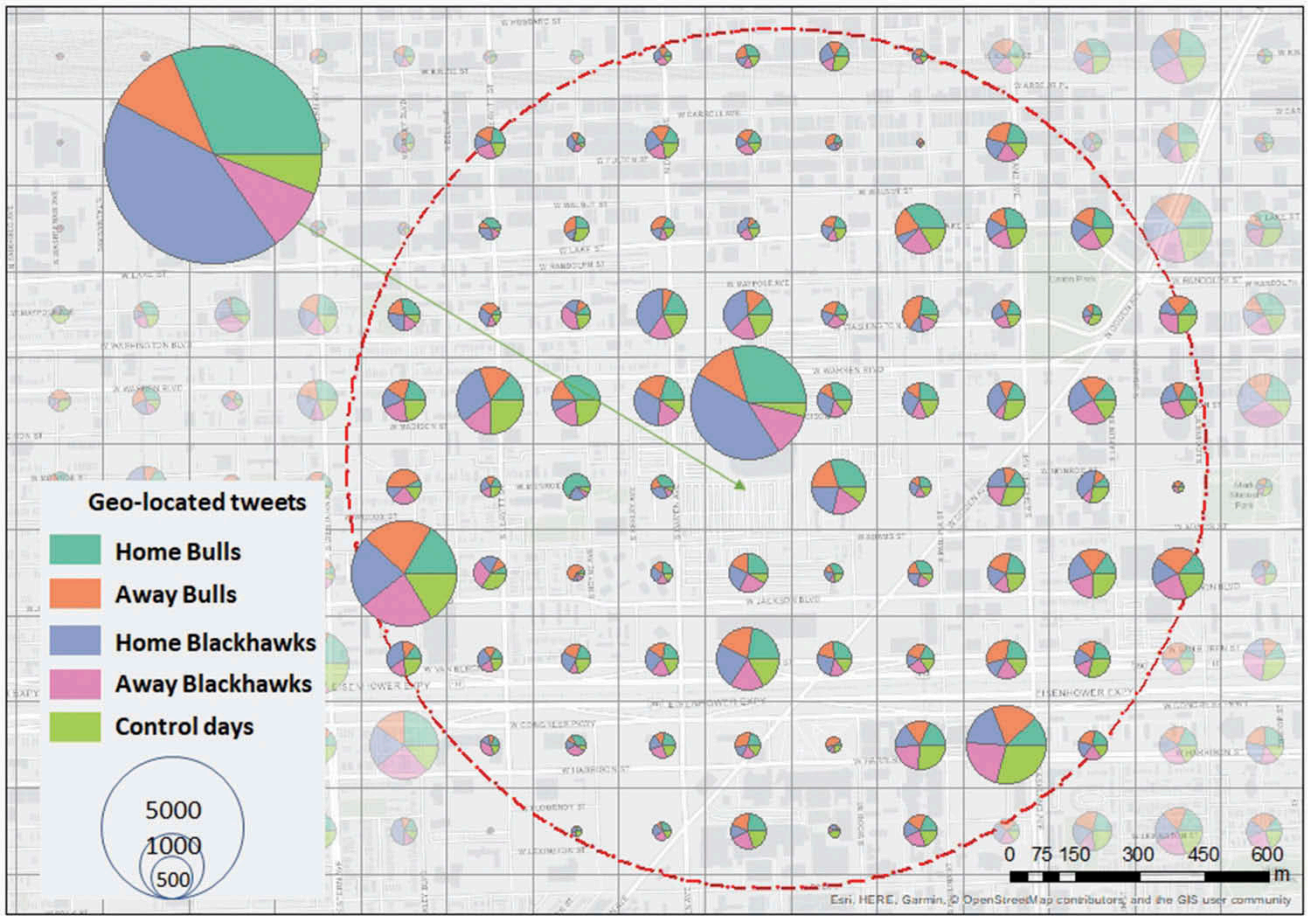
Density distribution of geo-located tweets around the venue; red dots circle is the 1km buffer around the venue.

Interestingly, while performing spatial density analysis on geo-located tweets (Figure 6) around the stadium, we noticed that, in the grid cells containing the stadium almost 43% of the tweets were written during home games of the Blackhawks (i.e. 5,066 tweets). About 32% of the tweets were written during home games of the Bulls (i.e. 3,717 tweets). During away games, the density of tweets was similar, with 1,117 tweets for Blackhawks games and 1,280 tweets for Bulls games; just 691 tweets were posted on the control days. All these tweets are extracted from the grid cell where just one crime was reported in Figure 5, supporting the information that coordinates for the open crime data are geomasked (i.e. crime incident locations are moved from incident location to random points within the crime street segments or blocks). During control days, the arena might hold other types of events during which people post tweets. However, these other events, such as concerts or circus shows, have not been previously linked to crime occurrences. In one grid cell southwest of the stadium, a large volume of tweets was observed, somewhat evenly distributed across the five bins. Strikingly, the location matches with the crime locations were also almost evenly distributed (see Figure 5). In the rest of the area, tweeting behavior tends to have a similar distribution across the bins.

**Figure 7.**
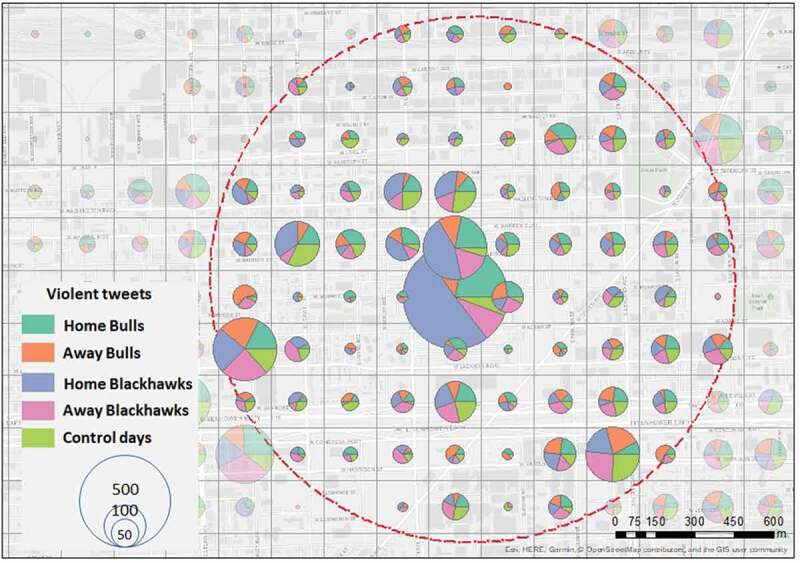
Density distribution of violent tweets around the venue; red dots circle is the 1km buffer around the venue.

Finally, we repeated the spatial density analysis with violent tweets. In Figure 7, we observed a higher number of violent tweets in the arena grid location (i.e. 51% for Blackhawks home games and 22% for Bulls home games). In contrast, during away games and control days, less than 10% of violent tweets occurred in the same place. On the west side of the stadium, in many grid cells we noticed a similar distribution between the five bins. This shows that Twitter data is valuable for detecting the occurrence of a public event based on a higher number of messages and whether the writing tends to be offensive or not. Violent tweets are almost equally distributed in the southwest part of the stadium with a high volume of tweets and crime. While 43% of the geo-located tweets in the stadium area were written during Blackhawks games, they account for 50% of violent tweets.

**Figure 8.**
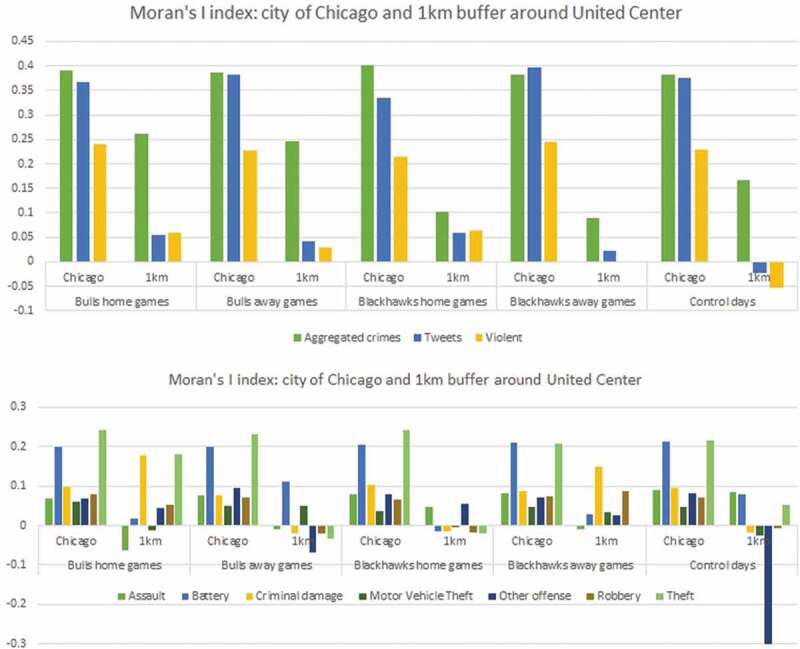
Moran’s I index for aggregated and disaggregated crime types, tweets and violent tweets; unit of analysis is the city of Chicago and a 1km buffer around united center.

Next, we performed a spatial autocorrelation analysis using Moran’s I Index (Figure 8). Aggregated crimes showed a moderate positive correlation with the highest value for Chicago Blackhawks home games. In all five bins, crime occurrences had an index between 0.382 and 0.401. This means that the cell density values for crimes were positively associated with each other throughout the study area. The spatial weight matrix needed for calculating the Moran’s I index is based on a first order queen contiguity. Interestingly, when applying the same index to the 1 km buffer around the stadium, the average for the five bins was 0.177, with the highest values seen during home and away games played by the Bulls, with values of 0.262 and 0.247 respectively, while during the Blackhawks home and away games the values were lower (0.101 and 0.088, respectively). While battery and theft showed a significant ~0.20 Moran’s I index in the city boundary, other crime types did not reach values over ~0.10 (Figure 8). There is low variability between the five categories. When analyzing the 1km buffer zone around the stadium, criminal damage and theft had a value of ~0.17 during the Chicago Bulls home game days, while the other categories had low values, under 0.05 (except for criminal damage during Blackhawk away games). All the other crime types generally had values under 0.10, while some are even negative. The crime type ‘other offense’ had a negative spatial autocorrelation of −0.334 during the control days, which differed significantly from game days, for which the value was around 0.

Geo-tagged tweets showed a moderate positive spatial autocorrelation, with an average of 0.371 and the highest value of 0.3965 during Blackhawks away games. Tweets in the 1 km radius were not clustered in any of the five bins and had an average of 0.030 Moran’s I index (0.058 for home games of the Blackhawks). The average Moran’s I for violent tweets was 0.231 with a low to moderate spatial clustering for the five bins. Moran’s I values were slightly higher during home games of the Bulls and away games of the Blackhawks, with values of 0.240 and 0.245, respectively. This suggests that offensive messages tend to be clustered across the city. There was almost no correlation around the stadium, with the highest values occurring during home games of both teams (0.060 and 0.064). Overall, the violent tweets tended to be less clustered than the geo-located tweets, while crimes and geo-located tweets had similar Moran’s I.

### 4.2. Spatial crime prediction

Our framework considered four comparison days, so 26 days were available for training prior to the last four days (part of the second sporting season). We trained six prediction models that are described in detail in Section 3.2.2, for which we split the 26 available days into two equal parts. The first part contained a lag of 13 days (i.e. featured values for training points selected mostly from the first season). Feature values for the prediction day were selected from the next 13 days, which fell mostly into the second season, except for the last game. This approach generated a single prediction for each of the seven crime types for each of the six types of models.

**Figure 9.**
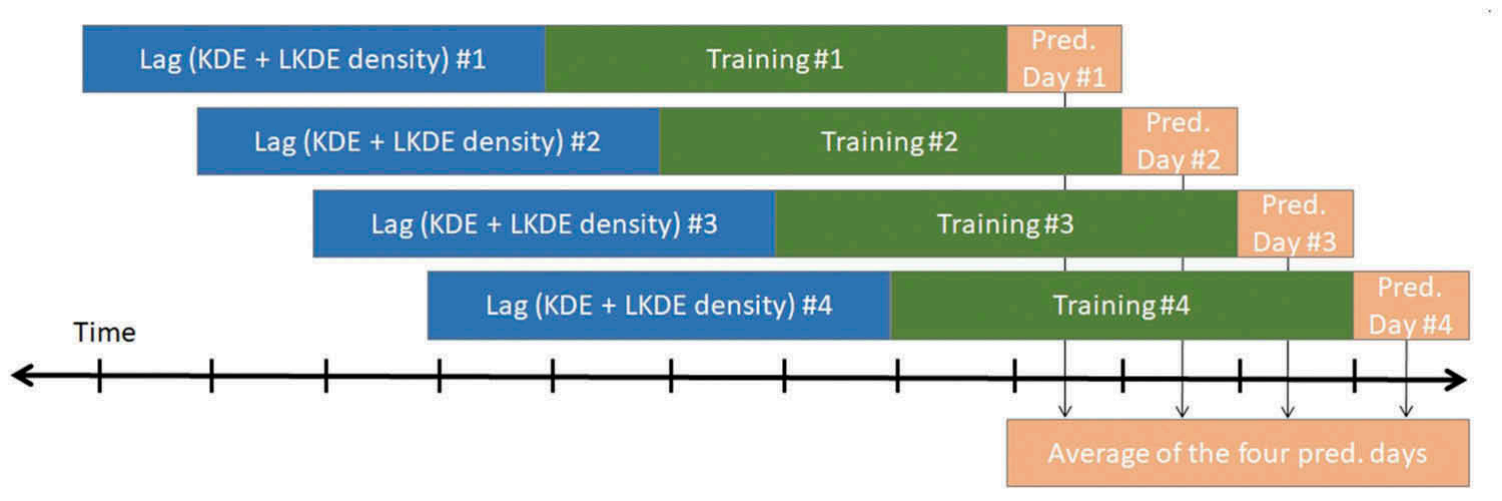
The sliding window prediction approach.

We follow a sliding window approach for the last four days of each time period in order to achieve a more generalizable prediction (Figure 9). Due to the limited days in the analysis, we considered just four prediction days. If we made a prediction only for the last day of the championship, the results might be skewed by different factors, which may be mitigated by averaging four different results. In this study, we considered the temporal path for the 30 days (e.g. the last four days will be in March and/or April when the second championship for both of the teams is coming close to an end). The results might still be skewed and randomizing the selection of training and prediction days would obtain different results. However, this research was beyond the scope of the current study. In section 3.2.2, 840 scenarios were mentioned that we calculated in this case study. The values for the four prediction days were averaged (Figure 9). For instance, if we were interested in the prediction models for Chicago Bulls home games, the first window would use the prediction day 03/11/2014, the second window would use the prediction day 03/13/2014, the third window would use the prediction day 03/17/2014, and the forth window would use the prediction day 03/22/2014 (these are the last four days when the basketball team played in 2014). Thus, the four AUC values were averaged in order to give a more reliable estimate than using just one day. The results were twofold: First, we compared crime types and temporal bins. Second, we compared the game days and comparison days.

#### 4.2.1. AUC comparison between crime types and the five temporal bins

We present our model evaluations in Section 3.2.2 covering 840 scenarios (six models, four predictions days, five bins, and seven crime types). We present the averaged AUC value between the four prediction days. In terms of the differences between the four prediction days, we calculated the standard deviation for each crime type in each of the bins. The values were generally under 0.05, so we will discuss only the aggregated values. However, we will consider researching the prediction aggregation effect in future work. AUC showed an improvement in all crime types by adding social media data.

**Figure 10.**
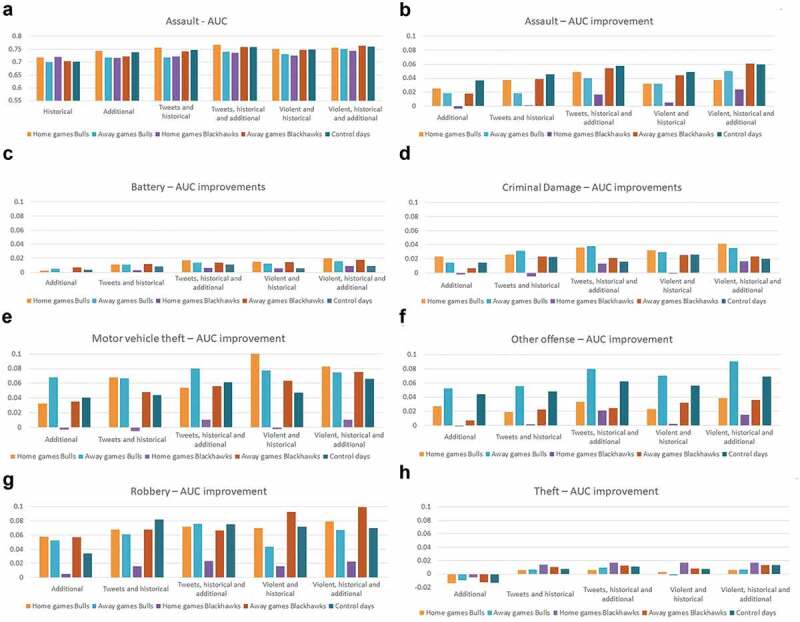
AUC and AUC improvement for the seven crime types *AUC real values are presented only for assault in order to save space.

Figure 10 shows bar charts for the AUC improvement of each crime type. For assault, the figure also shows the real values of the AUC. Generally, the AUC values ranged between 0.70–0.76 for assault, 0.74–0.79 for battery, 0.65–0.70 for criminal damage, 0.60–0.74 for motor vehicle theft, 0.65–0.77 for other offense, 0.65–0.79 for robbery, and 0.72–0.77 for theft. For all crime types, we noticed that the highest improvements occurred for the models including (1) tweets, historical and additional model and (2) violent, historical and additional model. Assault showed the highest values during Bulls home games; battery showed the highest values during Blackhawks away games and on control days; criminal damage showed similar values over the bins; motor vehicle theft had the highest values during Blackhawks and Bulls home games; other offense had the highest values during away games of the Blackhawks; robbery had higher values during Blackhawks home and away games and on control days; and theft showed similar values over the bins. It is interesting that motor vehicle theft had a 10-percentage point improvement in AUC for Chicago Bulls home games. During Blackhawks home games, the violent tweets model performed worse than the historical crime model. In addition, regarding the AUC values, the highest values, above 0.70, occurred during Blackhawks home games.

On control days, assault prediction was improved by at least 4 percentage points through adding tweets or violent tweets in the model. For assault, the away Blackhawks game days and control days had the highest AUC differences between bins. For criminal damage, the Bulls home and away games had a higher AUC increase. Other offense models had the highest AUC during Bulls away games, and almost all models improved for this bin by at least 6 percentage points. Robbery AUC improved by more than 8 percentage points by integrating tweets during control days and violent tweets for Blackhawks away games. Compared to the other crime types, battery and theft showed the smallest improvements in the AUC (i.e. under 2 percentage points). For theft, adding additional measures made the model performed worse than the historical crime model.

#### 4.2.2. AUC comparison between game and control days

**Figure 11.**
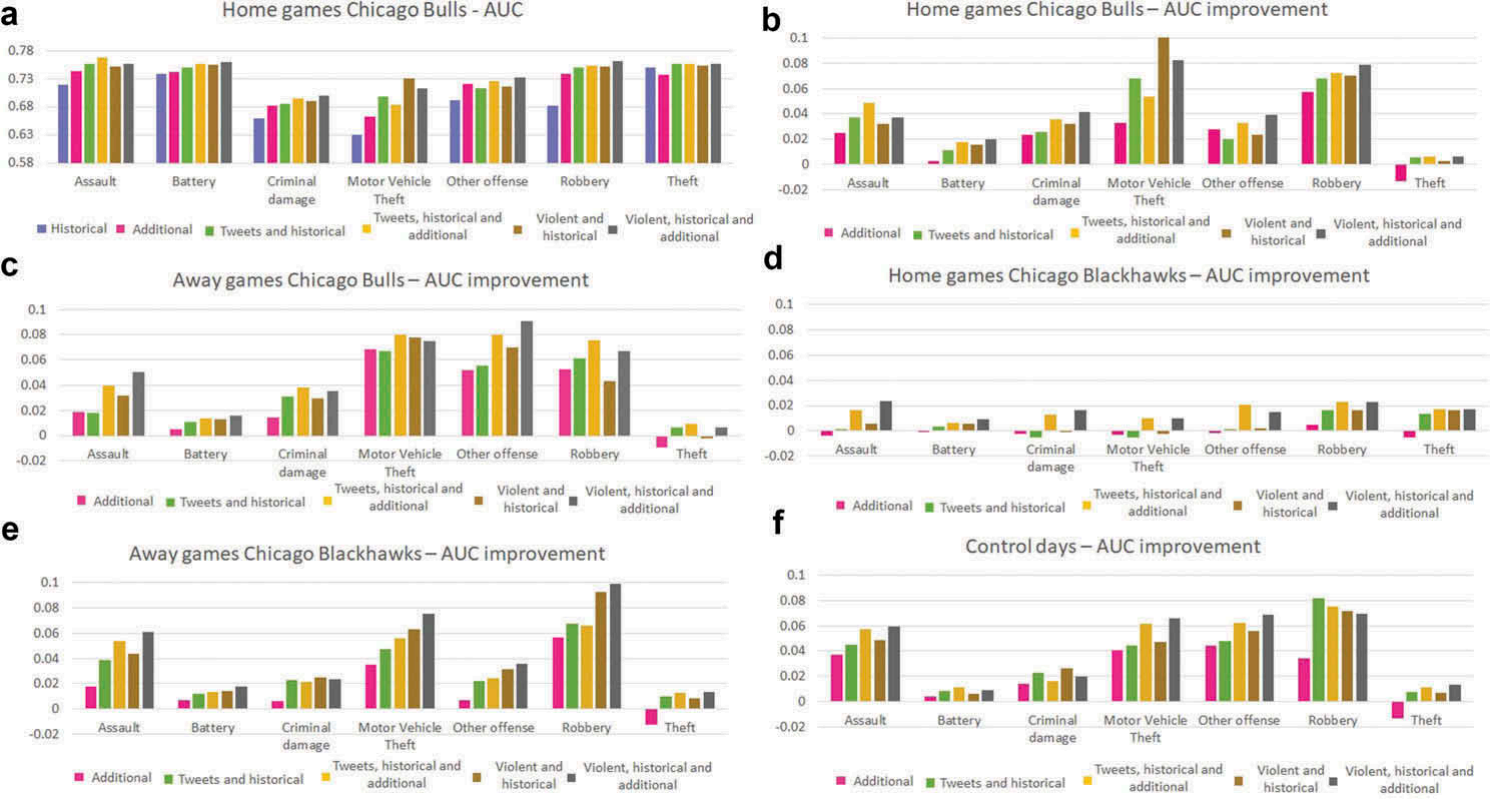
AUC and AUC improvement for the five bins *AUC real values are presented only for home games Chicago bulls in order to save space.

Because of the high attendance for sporting events, such as basketball and hockey games, plus additional people watching the games through other media sources at different locations throughout the city, we expected a change in spatial crime patterns and an increase in geo-located Twitter messages – mostly in the subsets of violent tweets. The density results indicated that spatial crime pattern changes between the five bins occur mostly during Bulls home games. There was a general slope pattern, the lowest AUC for the historical crime model, increasing systematically in the next five models (Figure 11).

For Chicago Bulls home games, the highest improvement occurred when adding tweets and violent tweets for motor vehicle theft, and then for robbery. These two crime types showed more than a 6 percentage points increase in the AUC. Theft and battery had low increase values (2 percentage points at the most), and the AUC for theft decreased by 2 percentage points when adding additional data. Major improvements for motor vehicle theft, other offenses, and robbery were shown for Chicago Bulls away games. The theft AUC decreased when adding additional data during home games.

While there were more tweets and violent tweets in the city during Blackhawks home games compared to other bins, the prediction models showed lower improvements than for other bins. The maximum AUC improvement in this bin was 2 percentage points for some models, while other models decreased in accuracy compared to the historical model. For Chicago Blackhawks away games, robbery and motor vehicle theft showed high AUC improvements while including violent tweets together with additional data (10 and 8 percentage points, respectively), followed by assault with a 6 percentage point improvement; theft, battery and criminal damage showed low improvements of 2 percentage points or less. While expecting lower improvements using tweets for control days, the models showed high improvements for assault, motor vehicle theft, other offense, and robbery.

## 5. Discussion

This study provides further insight into the spatial relationships between crime occurrences and geo-tagged Twitter messages. It also integrates demographic, socio-economic, and environmental information for the city of Chicago during basketball – Chicago Bulls – and hockey games – Chicago Blackhawks. Aside from discovering valuable spatial dependencies, this research adopts a localized kernel density estimation (LKDE) model for crime prediction during game and control days. We assume that some future crimes will occur in similar locations where historical crimes and historical tweets were posted, and our assumption was confirmed by higher prediction performance for models including social media data compared with traditional ones based only on historical crime. Yet, the models are applied for only seven crime types, each one of them including various subtypes that have diverse space-time patterns associated, implicitly routine activities. Whilst both property (motor vehicle theft, other offense) and violent crimes (assault, robbery) display gains in performance due to social media inclusion, this does not extend to every crime type (i.e. battery and theft). Cross-crime type interactions may be adding prediction power for crimes where historical data has clear space-time patterns.

### 5.1. Spatial distribution of crime occurrences and social media data

While analyzing aggregated crime types’ density for 1 km radius around the United Center, two highlights emerged: shifting crime hot spots in the immediate vicinity of the stadium (i.e. higher crime volume during home game days for both teams) and static crime hot spots in the western part of the stadium, supporting the notion that crime behavior shifted in space and time (Malleson and Andresen 2015a). The static hotspots can be linked to the highly clustered African-American population residing there, which, as shown in previous literature, may be correlated with higher crime rates (Quillian and Pager 2001, Gabbidon and Greene 2018). Encountering racial discrimination has the potential to increase African-American offending, also because of over-policing – a well-known phenomenon in the United States (Weitzer 2017). Other researchers suggest that higher poverty rates (in both predominantly white and black neighborhoods) might explain differences in crime rates rather than intuitions or assumptions about racial factors (Hannon and DeFina 2005).

Aggregated crimes and geo-located tweets form moderate spatial clusters in the city of Chicago, and violent tweets have a low positive correlation. We observed battery and theft to be more spatially clustered than all other crime types. Battery is a criminal offense that is lower in the violence ranking than assault, which involves offensive physical contact with another individual. In contrast, theft involves the illegal taking of another individual’s property. Assault, criminal damage, motor vehicle theft, robbery, and theft show a more spatially clustered pattern during game days. For some crime types, like theft or drug handling, a spatial link between networks of criminals exists. For example, when small crime clusters are identified in the field, they may represent acts of the same gang or group members in specific locations, such as the city of Chicago (Block 2000, Klein and Maxson 2010).

Geo-located Twitter messages and violent tweets had higher densities around the United Center. In the surrounding areas (1 km radius), the density is similar in all five bins. While analyzing the entire city, bivariate spatial autocorrelations between crimes and tweets show higher spatial clustering during days where the Chicago Bulls played a home game but not for the Chicago Blackhawks. This may indicate that there is more spatial connectivity between crime locations and the use of social media when the basketball team plays home games. This finding is consistent with the density analyses, in which a different crime count between home game days and comparison days is apparent. Fans can become violent or distracted after games, irrespective of whether a game is won or lost, causing violence and potentially traffic negligence (Smith 1979, Wood *et al*. 2011). Research shows that game outcomes can cause negative or disruptive behavior in sports fans (Andresen and Tong 2012, Copus and Laqueur 2014). However, analyzing the outcome of a game would imply sociological and psychological explanations, which was not the purpose of this study.

### 5.2. Covariates explaining crime occurrences

Many covariates need to be considered when analyzing crime occurrences and implementing crime prediction models. Usually, researchers tend to use enduring characteristics as covariates, such as demographics, socio-economics, and environmental factors (e.g. parking areas, lighting, bars, buildings). Our study supports recent research showing the importance of dynamic variables in crime prediction. For example, urban events, policy changes in the use of social media, new crime prevention models, neighborhood watch, highly changeable weather conditions, emerging events (e.g. protests, transport blockage, tourist seasons), and others constitute elements which show dynamic spatial and temporal characteristics. In this study, we used geo-located tweets and violent tweets as dynamic spatiotemporal data. Despite the fact that tweets are unlikely to explicitly detail the planning of a crime or its characteristics, messages which refer to various violent actions can be quantified. Although social media text analysis was still a challenge, we used a method of extracting an influential explanatory variable, namely violent tweets, through sentiment analysis and bag-of-words extraction. In order to extract the most important explanatory variables, we applied a Random Forest classifier with the dependent crime data.

As such, in our approach, we considered ‘violent’ tweet density and crime density, regardless of whether they referred to a specific crime or not. The 24 hours aggregation assumes crime and tweets occur at the same time interval, yet there is a chance that a group of tweets is posted before or after a crime event occurs. Most games happen in the (early to late) evening, so the time frame for tweets following a crime is short. Crime occurrences are sparse per location and time frame, hence by aggregating them per hour we may have too few points – at least for the way our models are built. Through the 24 hours aggregation, we assumed to have more ‘violent’ tweets during sporting events than during comparison days. However, while this is clear around the stadium, the pattern dissipates across the city. Crime prediction models show performance improvements while adding Twitter data not only for game days but also for control days, which supports the idea that social media plays a role in city-wide prediction – if only the area around the stadium is considered, the performance may or may not be higher during game days.

In addition, we used Twitter data in combination with ambient LandScan data to create a new variable, entitled ‘population at-crime risk,’ for each of the five bins. Although this feature showed high importance during Random Forest feature selection, there is literature investigating different ways of calculating the ambient population. However, to date, there is no unanimously accepted method in the field of spatial crime analysis for calculating whether a population is at risk.

### 5.3. Is twitter data influential for crime prediction models?

As explained before, a key contribution of this study was applying density and spatial autocorrelation techniques in order to show the spatial patterns of crime and social media on a buffer around both a venue and for the entire city, after which important explanatory variables for crime prediction models were selected.

The prediction models focused on KDE and LKDE density methods. KDE assumes that areas with historically high crime occurrences are more likely to encounter crime in the future. LKDE addresses two limitations of KDE, showing lower computational complexity and the kernel function choice. This method establishes a dynamic bandwidth and exponentially decaying interpolation kernel according to the data fed in the algorithm. Practically, the LKDE creates focused convolution kernels when dense data is available and enlarged kernels for spread data. One potential shortcoming of both methods could be that both begin by overlaying a grid (with *n* equally sized cells) on top of the study area. Current literature offers some guidance to determine grid cell size (Caplan *et al*. 2011, Kennedy *et al*. 2011), but there is no fully accepted rule about it. A density estimate based on the center points of each grid cell was calculated, and thus changes in this parameter would probably yield different results. There are various ways to approximate an integral value for a grid square (e.g. by using multiple points in a grid instead of the center). We acknowledge that by using a different approach than the center point, which might produce different results. Ultimately, building the logistic regression models using a LibLinear classifier started once the density estimates were obtained. For future work, we are planning to test multiple classifiers.

By integrating geo-located tweets and violent tweets into prediction models we noticed various improvements. The outcomes were highly dependent on historical data that was fed into the prediction model. The predicted areas represented an extrapolation from past crimes, so they were highly dependent on the quality of the historical crime data. Biases introduced in the training data will likely skew the prediction outcomes. For example, if a specific pattern is shown in the training data, the model will most likely predict according to that pattern. Thus, if the prediction day is an outlier of the pattern, the outcome will show inaccurate values. At the same time, an increase in social media messaging or violent posts when crime is stable can also modulate the prediction. Thus, historical crime and Twitter datasets present limitations, which resulted in a ‘relative prediction improvement.’ Nonetheless, our results, complemented by previous literature, indicate the feasibility of using Twitter data in capturing irregular routines, and thus crime.

As a follow-up, historical data can often be a cause of inconsistency in the prediction values while analyzing Chicago Blackhawks home games. The maximum AUC improvement in this bin was 2–3 percentage points, while in the other bins we found values that improved by 6–8 percentage points. In addition, some of the models were worse than the historical crime prediction. Considering the volume, more tweets and violent tweets were posted in this bin, but they were not able to exhibit significant increases in the AUC. While testing other prediction days for Blackhawk home games, we noticed a greater improvement than the one discussed in this study for the averaged four days. It could be that information from the explanatory variables showed inconsistency for the prediction days or those other elements should have been considered for the prediction days (e.g. weather conditions, higher attendance at the stadium, other events in the city, policy change). A more detailed analysis is needed from criminological and sociological perspectives to determine the sources of these inconsistencies.

We expected higher AUC during game days in models including tweets and violent tweets because more individuals use social media messages to express themselves about sports, as past literature has already shown (Corney *et al*. 2014). Interestingly, we noticed that our models also improved for control days. This highlights the importance of considering the prediction day and environmental factors in an analysis, as their integration can change the prediction patterns observed. The results were for the entire city of Chicago, so these results might also show that regardless of whether there is a game at the stadium, integrating tweets and mostly violent tweets were able to improve prediction models. While behavioral criminology and spatial crime studies show that changes in crime occur around a stadium when home games are played, these results for the entire city show prediction improvement for all the selected days.

Overall, Twitter data can capture routine activities associated with crowd-based events in a way that would be impossible without an advanced data infrastructure that can capture micro-level population changes. Compared to other dynamic ‘big data’ sources recently introduced in crime prediction models, Twitter data is highly accessible and includes a wide level of detail (personal, temporal and content-wise).

### 5.4. Crime types space-time patterns: distinct prediction outcome

Regarding prediction for crime types, distinct improvements were seen after introducing social media variables. Battery and theft, which had the highest density in our dataset and were among the most spatially clustered crime incidents, yielded the lowest model improvements. This shows that when the crime data is dense and widely spread over the prediction territory, together with spatiotemporal stability, additional data will influence the prediction but not as much as it influences less dense crime types. These crime types can also have temporal patterns that predict activity in the immediate feature, such as repeats and near repeats (Chainey 2012), so additional data could harm the accuracy of the prediction. While, in general, tweets and violent tweets improved prediction accuracy compared with the historical prediction models, other offenses and motor vehicle theft were identified to be the main crime types for which the Twitter data had the highest influence in prediction models.

For a venue with diverse visitors for sporting events in a given time interval, correlation with some specific crime types occurs because of the presence of victims and offenders together in a greater concentration. Large events attract high attendance and they change the normal cycle of activities in an area, thus representing an irregular routine, an event-routine activity for a location that can attract or generate criminal behavior. Twitter data capture potentially criminogenic movement patterns, which are difficult for authorities to extract from other data sources: individual personal information, where people are located, what are their feelings, what are their plans and other information.

Motor vehicle theft is a property crime and it refers to the theft or attempted theft of a motor vehicle (Federal Bureau of Investigation 2018). This crime type has a strong seasonal trend and is one of the most commonly occurring crimes in the USA, with 689,527 reported incidents nationwide in 2014 (Piza *et al*. 2017). People may offer opportunities to offenders by parking their cars in low-security parking areas and also by leaving valuables in the car, which can serve to generate crime (Brantingham and Brantingham 1981, Kinney *et al*. 2008). Furthermore, the car model and the low lighting may be a risk factor for motor vehicle theft (Clarke 2002). Twitter data offers a unique opportunity to measure how many people, and by proxy cars, are in an area at a specific time. Moreover, at the individual level, statistical and graph analysis can be applied to classify user profiles based on suspicious tweets and identify influencers’ background to find connections with criminal activity. For this study, we are not approaching the individual level, yet it is important to mention the important role played by Twitter analysis.

Compared with theft and battery, which are dense and show clear spatial clusters, motor vehicle theft is scarce, revealing the need for additional information in understanding it. This study shows that Twitter data captured part of the content needed for predicting vehicle theft in space and time. Future research should consider additional explanatory variables for this crime time in order to understand it better.

Another property crime, criminal damage, showed the highest improvements when adding violent tweets and additional data in the models for Chicago Bulls home and away games. Criminal damage occurs when someone willfully destroys or damages property without the consent of the owner (Federal Bureau of Investigation 2018). This crime type typically refers to property damage such as vandalism, damage to a vehicle, or damage to state property.

The other offense crime type is a complex amalgamated category, and it can include telephone threat, possession of burglary tools, harassment by telephone, violation of order or protection, other crimes against a person, other crimes involving property, compounding a crime, and others. This type of crime can happen in the awareness space of a person that can overlap with the action space of a possible offender (Brantingham and Brantingham 1981). In addition, it can represent hooliganism behaviors, and such events might be associated with irregularly large groups of people that are not associated with traditional correlates of crime. Examining basketball fan behavior during home and away games may prove to be an interesting sociological study, similar to football hooliganism studies in the United Kingdom (Dunning *et al*. 2014), where stadiums act as crime attractors and generators (Brantingham and Brantingham 1981).

### 5.5. Limitations

An important limitation concerns the modifiable areal unit problem (MAUP) (Openshaw and Openshaw 1984). The spatial units used in this study were squared grid cells of 200m x 200m over Chicago, which can offer an overview of crime distribution at this scale. However, while changing the scale, and shape, we would likely have found different results. For example, grid cells that are considered to be crime hot spots can easily become neutral. This is very important for law enforcement and can lead to inaccuracy in crime prediction, and, ultimately inappropriate spatial assignment of police patrols. In addition, we acknowledge the problem of temporal aggregation in 24 hours slots, which is similar to the spatial aggregation discussed in the MAUP. While for this research we obtained daily predictions with daily general patterns, for a real-time crime prediction model, the design needs to be changed to other temporal aggregation. This brings ideas for future research: different aggregation bins, moving average, times of the day, and others.

Another concern is related to crime data quality and geo-privacy aggregation, namely at the street level or block level. Practically, if the real coordinate for a crime occurrence is 50-100m away from the analyzed location, then the analysis is just relative. Crime locations are extracted from freely available data made available by the Chicago Police, with unreported crimes not represented in this study. In addition, the temporal stamp of some crime types is unclear, adding additional uncertainty in the analysis. This limitation is widely-known to criminologists and other researchers using crime data. Therefore, when using limited crime data and publicly available subsets of Twitter data, we need to be careful in assessing the usefulness of social media data for crime prediction (e.g. the results are relative, and they can be skewed from both sides – for both crime and tweets). The methods presented in this paper can be used with real locations if available, and it would be interesting to see the change in patterns and the effects of spatial and temporal aggregation using the most accurate data.

## 6. Conclusion

Findings from this study suggest that using Twitter data can have a significant influence on building predictive models for seven crime types, when used in conjunction with their time stamps, into spatial prediction models. Our study reinforces the strength given by learning about individuals and groups from only analyzing their behavior in social media, allowing the integration of human mobility (where people are at certain times) for possible improvement of space-time crime prediction. Injecting tweets or violent tweets into prediction models led to an improved prediction accuracy of crime occurrences compared to a prediction where only historical crime data was considered.

The results of this work, supplemented by additional research, can be helpful in understanding the spatial distribution of criminal activity during basketball and hockey games and the usability of implementing geo-coded social media data (and violent tweets) on predicting future crimes. The outcomes of this study point to several future paths, including (1) further tailoring of social media text analysis for extracting significant features, include message topics, helping to extract pre- and post- crime messages; (2) incorporating additional dynamic spatial data (mobility data, mobile phone data) in crime prediction models and extracting possible crime risk factors; (3) exploiting the results to incorporate information about public events in the city in a single prediction model that can be robust and transferable. The current research can be applied to other types of events and to other locations. In addition, it is worth mentioning the necessity of studying more prediction evaluation methods. In this analysis, we used the surveillance plots, which measure the percentage of true incidents captured on the prediction day that occurred within the percentage of the area surveilled, for which we presented a summary of their AUC.

In future work, we are planning to analyze different outcomes when covering different percentages of an area. Moreover, we will investigate more evaluation metrics, such as the ones proposed by Adepeju and colleagues (2016). Note that accurate predictions do not automatically lead to major crime reductions, but they can be relevant in decision-making. However, results may vary because of the geographical space, along with culture, religion, education, socioeconomic factors, and human behaviors that are different across the globe.
